# Embracing the future—is artificial intelligence already better? A comparative study of artificial intelligence performance in diagnostic accuracy and decision‐making

**DOI:** 10.1111/ene.16195

**Published:** 2024-01-18

**Authors:** Ângelo Fonseca, Axel Ferreira, Luís Ribeiro, Sandra Moreira, Cristina Duque

**Affiliations:** ^1^ Neurology Department Hospital Pedro Hispano, ULS‐Matosinhos Matosinhos Portugal

**Keywords:** artificial intelligence, automated diagnosis, automated treatment, ChatGPT, clinical reasoning

## Abstract

**Background and purpose:**

The integration of artificial intelligence (AI) in healthcare has the potential to revolutionize patient care and clinical decision‐making. This study aimed to explore the reliability of large language models in neurology by comparing the performance of an AI chatbot with neurologists in diagnostic accuracy and decision‐making.

**Methods:**

A cross‐sectional observational study was conducted. A pool of clinical cases from the American Academy of Neurology's Question of the Day application was used as the basis for the study. The AI chatbot used was ChatGPT, based on GPT‐3.5. The results were then compared to neurology peers who also answered the questions—a mean of 1500 neurologists/neurology residents.

**Results:**

The study included 188 questions across 22 different categories. The AI chatbot demonstrated a mean success rate of 71.3% in providing correct answers, with varying levels of proficiency across different neurology categories. Compared to neurology peers, the AI chatbot performed at a similar level, with a mean success rate of 69.2% amongst peers. Additionally, the AI chatbot achieved a correct diagnosis in 85.0% of cases and it provided an adequate justification for its correct responses in 96.1%.

**Conclusions:**

The study highlights the potential of AI, particularly large language models, in assisting with clinical reasoning and decision‐making in neurology and emphasizes the importance of AI as a complementary tool to human expertise. Future advancements and refinements are needed to enhance the AI chatbot's performance and broaden its application across various medical specialties.

## INTRODUCTION

The concept of using computers to simulate intelligent behaviour and critical thinking was first described by Alan Turing in 1950 [1]. In the book *Computers and Intelligence* Turing described a simple test, which later became known as the Turing test, to determine whether computers were capable of human intelligence [2]. Six years later, John McCarthy described the term artificial intelligence (AI) as ‘the science and engineering of making intelligent machines’ [3, 4].

Artificial intelligence (AI) began as a simple series of ‘if, then’ rules and has advanced over several decades to include more complex algorithms that perform similarly to the human brain [5]. By harnessing the power of machine learning and data analytics, these algorithms can now analyse vast amounts of medical data, uncover patterns and generate valuable insights. AI has emerged as a promising technology with vast potential to transform the healthcare industry [6]. Disease diagnosis and treatment has been a focus of AI since at least the 1970s, when MYCIN was developed at Stanford University for identification of blood‐borne bacterial infections [7]. Although these systems showed promise for accurately diagnosing and treating diseases they were not substantially better than human diagnosticians, and they were poorly integrated with clinician workflows and medical record systems [7, 8, 9, 10, 11, 12].

In the context of natural language processing (NLP), IBM created an open‐domain question‐answering system in 2007, named Watson, that used various searches to analyse data over unstructured content to generate probable answers [13]. These developments led to IBM Watson's victory on Jeopardy in 2011, still being a landmark for the AI field. In 2015, a team of researchers from Stanford University developed the transformer architecture; in 2018, OpenAI released GPT‐2, a large language model (LLM)—machine learning algorithms trained on vast amounts of text—that could generate realistic and coherent text, followed by GPT‐3 in 2020. More recently, a vast amount of LLMs has been created with different numbers of parameters, such as GPT‐4, PaLM 2, Claude v1 and Cohere. All these advancements led to superior performance in language tasks, bridging the gap between human−computer interactions and making AI systems more adaptable and integrated into real‐world applications. The result has been a surge in AI capabilities, from chatbots and voice assistants to advanced text analytics and generation [14]. ChatGPT is amongst the most widely used AI chatbot. It makes use of the Generative Pre‐trained Transformers (GPTs) autoregressive transformer model that uses 175 billion parameters [14]. This generation of LLMs has demonstrated exceptional performance in a wide range of NLP tasks, including language translation, summarization and question‐answering. The transformer‐based architecture is one of the latest breakthroughs in NLP and introduced an attention mechanism that allows contextual relationships between words/tokens in a sentence to be captured [15]. This attention mechanism enables transformers to consider the entire input sequence at once, addressing the limitations of previous models that processed text sequentially. The training procedure is composed of two main steps: (1) pre‐train using a large set of corpora such as Wikipedia, scientific journals and news articles, where the main task is to predict the next word in a sentence; (2) smaller and human‐made examples are demonstrated as correct behaviour based on reinforcement learning using human feedback making use of proximal policy optimization as the learning method [15].

By drawing information from a patient's electronic medical record and other electronic resources, this technology could be applied to provide evidence‐based medicine responses. As such, it opened new possibilities in evidence‐based clinical decision‐making [6, 13, 16]. In 2017, Bakkar et al. [17] used IBM Watson to successfully identify new RNA‐binding proteins that were altered in amyotrophic lateral sclerosis.

Given this momentum, along with improved computer hardware and software programs, digitalized medicine became more readily available and AI applied to medicine started to grow rapidly. In June 2023, an article published by Bitkina et al. [18] provided an overview of the current state of artificial intelligence in the field of medical technologies, reviewing 89 papers. Eighty‐six of these were published after 2011. The main area of AI application in medicine was oncology (55%), and in sixth place was neurology with 3% published papers on this topic, mainly focusing on neurodegenerative diseases.

Since its launch in November 2022, ChatGPT has been widely used by individuals for recreational and professional purposes. Many studies have suggested its potential application in the medical field, including in patient diagnosis and medical education, but major limitations and ethical considerations still exist [19].

In 2020, the American Academy of Neurology (AAN) released the Question of the Day application [20] as an educational tool for neurologists and neurology residents. These are multiple choice questions that are created for neurologists and follow a curriculum based on the American Board of Psychiatry and Neurology Maintenance of Certification content outline. Each question describes a real‐life scenario involving a patient presenting with specific symptoms or clinical findings. The questions require careful interpretation of provided information, knowledge about the underlying condition, diagnostic tests and treatment options in order to select the correct answer. They are two‐step questions consisting of clinical vignettes designed to assess the ability to interpret symptoms and to arrive at a diagnosis and/or appropriate management strategy, such as the best diagnostic test or treatment. The two steps required for each question include (1) interpretation of the clinical vignette to reach a diagnosis (this is an implicit step, but no answer is required); (2) correctly answer the multiple choice question (i.e., the most appropriate treatment). Only one choice is correct for each multiple‐choice question. A recent article has estimated that, in 2022, the total number of users was 11,000 per year, with average daily users of 1500 from 123 countries [21].

### Aim

The aim was to explore the reliability of LLM‐based AI software in healthcare clinical reasoning, diagnostics and decision‐making.

## METHODS

### Study design

**FIGURE 1:**
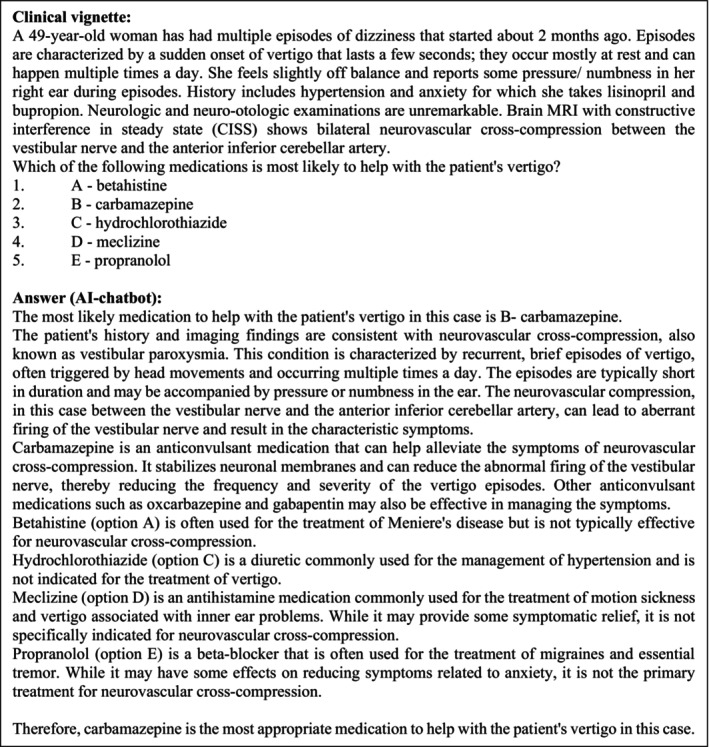
Example of a clinical vignette and the corresponding answer. Category: Neuro‐ophthalmology/neuro‐otological disorders. Published 28 March 2023.

**FIGURE 2:**
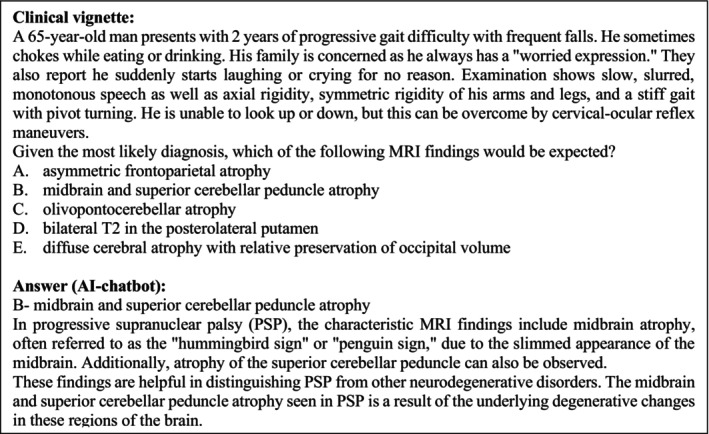
Example of a clinical vignette and the corresponding answer. Category: Movement disorders. Published 25 February 2023.

**FIGURE 3:**
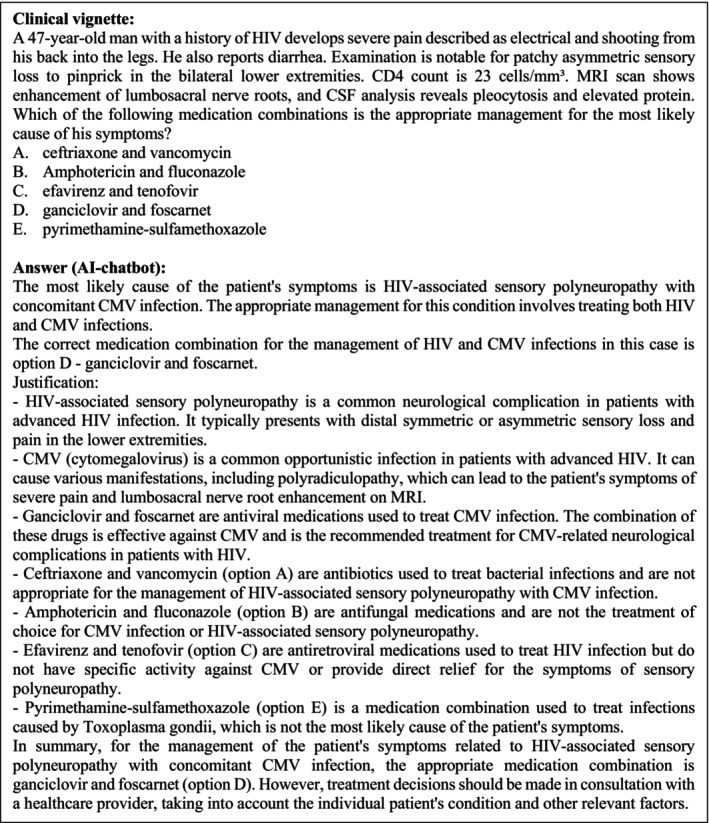
Example of a clinical vignette and the corresponding answer. Category: Neuroinfectious diseases. Published 6 December 2022.

**FIGURE 4:**
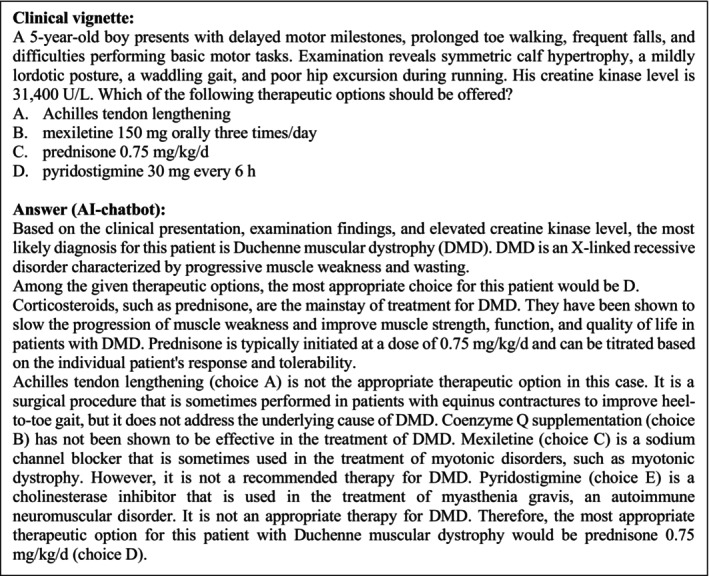
Example of a clinical vignette and the corresponding answer. Category: Neuromuscular diseases/disorders. Published 24 March 2022.

**FIGURE 5:**
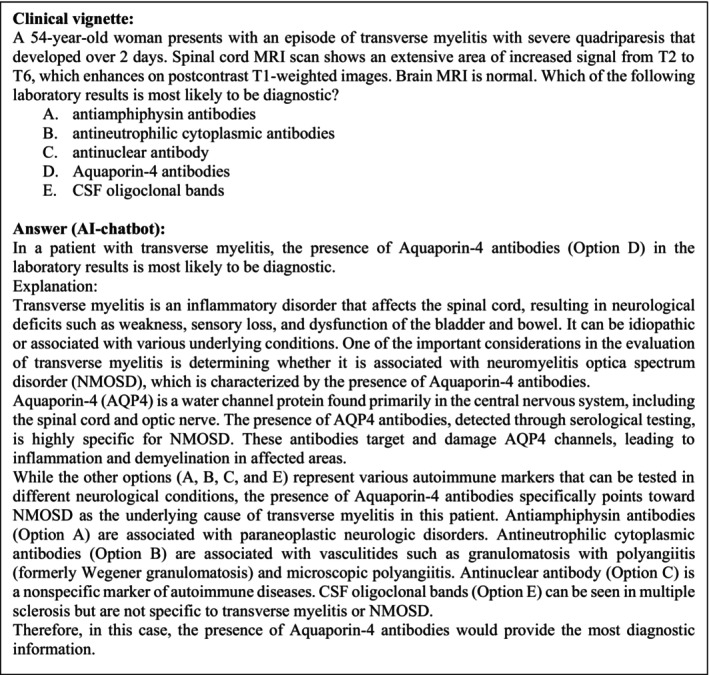
Example of a clinical vignette and the corresponding answer. Category: Brainstem and spinal cord syndromes. Published 22 August 2022.

This was an exploratory cross‐sectional observational study. A pool of questions in the form of clinical cases with multiple choice questions from the Question of the Day application (which provides daily board‐examination‐style multiple choice questions) was used. Topics include a wide variety of neurology subspecialties that are divided by the app into 22 categories. LLM‐based AI software (AI chatbot) responses were generated by ChatGPT (GPT‐3.5 version) and neurology peers’ response data (relative frequency of right answers) were provided by the app. Examples of questions (followed by ChatGPT's answers) are provided in the Results section (Figures 1, 2, 3, 4, 5).

### Primary end‐point

The primary end‐point was established as the mean success rate, defined as the mean percentage of correct answers per category to multiple choice questions. The objective was to assess the reliability of ChatGPT (GPT‐3.5 version) in correctly interpreting neurology‐related clinical vignettes and answering complex multiple choice questions, compared to neurology peers.

### Secondary end‐points

The first secondary end‐point was the success rate per category, defined as the percentage of correct answers per category. This secondary end‐point measures ChatGPT's accuracy across distinct categories, benchmarked against neurology peers.

The second end‐point was correct justification, intended to validate the correct answers given by the AI chatbot in order to exclude luck/randomness by asking for the justification for its correct answers. For this end, two investigators simultaneously and blind to each other's decision read the justifications and decided whether they were adequate or not. When an immediate agreement was not reached, the case was discussed with a third investigator and the decision was based on the consensus of two out of the three investigators.

The final end‐point was the correct diagnosis, designed to assess ChatGPT's capability to deduce the correct diagnosis from clinical scenarios when the diagnosis is not explicitly mentioned in the clinical vignette. This was provided within the ‘justification’ section in the AI chatbot's answer.

### Data analysis

The results were obtained using answers from AI chatbot by entering the following prompt: ‘answer the following multiple choice question about neurology’ (in order to assess the primary end‐point [success rate] and a secondary end‐point [success rate per category]) ‘and justify’ (to assess the remaining secondary end‐points). The answers were obtained for 10 questions per category, except when there were fewer than 10 questions available, in which case all the available questions were used. The questions were randomly chosen based on the most recent publication since 2020 until 30 April 2023. The questions requiring only the interpretation of a provided image were excluded.

The percentage of correct answers was calculated for each category (‘success rate per category’) and the primary end‐point (‘success rate’) was calculated using the mean percentage of correct answers per category (Table 2). The mean success rate per category of peers was obtained by the mean percentage of the peers' right answer per category (this information is provided by the app for each question, i.e., for question *x*, 60% of peers answered correctly). A comparative analysis between the ‘success rate’ of the AI chatbot and human experts was then conducted using *t* Student paired test, after employing the Shapiro–Wilk test and visual inspection of histograms to assess data normality. Data were then paired by category to compare success rate per category between the AI chatbot and peers.

## RESULTS

A total of 188 questions were included across 22 different categories, namely (1) normal structure process and development through life cycle; (2) anatomy/localization; (3) ethics/professionalism; (4) autonomic nervous system disorders; (5) brain and spinal trauma; (6) sleep disorders; (7) brainstem and spinal cord syndromes; (8) epilepsy; (9) metabolic diseases, nutritional deficiency states and disorders due to toxins, drugs and physical agents; (10) neuro‐ophthalmology/neuro‐otological disorders; (11) psychiatric disorders; (12) neuromuscular diseases/disorders; (13) genetic and developmental disorders; (14) neuro‐oncology/neuro‐oncological disorders; (15) headaches and pain disorders; (16) movement disorders; (17) neurology of systemic disease; (18) vascular disorders; (19) behavioural and neurocognitive disorders; (20) cranial nerve palsies; (21) neuroinfectious disease; (22) neuroimmunological and paraneoplastic disorders of the central nervous system and demyelinating disorders. Categories (1)−(4) and (20) had fewer than 10 questions each, and hence only three, one, three, nine and two questions respectively were included. Two questions had been excluded because the answer relied on image interpretation. In Figures 1, 2, 3, 4, 5 examples of the questions and respective AI chatbot answers are provided.

The results obtained by the AI chatbot are presented in Table 1. Results obtained per category and the overall performance of the AI chatbot and neurology peers are shown in Table 2. For the primary end‐point analysis, the AI chatbot achieved a mean success rate of 71.3% (standard deviation of 19.1). The difference between the AI chatbot and neurology peers was not statistically different (71.3% vs. 69.2%; *t*(21) = 0.634; *p* = 0.533).

**TABLE 1:** AI chatbot results.

Category	Correct answer *n* (%)	Correct diagnosis *n* (%)	Adequate justification *n* (%)
Normal structure process and development through life cycle	3/3 (100)	3/3 (100)	2/2 (100)
Anatomy/localization	1/1 (100)	1/1 (100)	1/1 (100)
Ethics/professionalism	3/1 (100)	3/3(100)	3/3 (100)
Brain and spinal trauma	9/10 (90)	10/10 (100)	9/9 (100)
Sleep disorders	9/10 (90)	9/10 (90)	9/9 (100)
Autonomic nervous system disorders	7/9 (77.8)	3/4 (75)	6/7 (85.7)
Brainstem and spinal cord syndromes	8/10 (80)	8/8 (100)	7/8 (87.5)
Epilepsy	8/10 (80)	9/10 (90)	9/10 (90)
Metabolic diseases, nutritional deficiency states and disorders due to toxins, drugs and physical agents	8/10 (80)	8/8 (100)	8/8 (100)
Neuro‐ophthalmology/neuro‐otological disorders	8/10 (80)	7/9 (77.8)	6/8 (75)
Psychiatric disorders	8/10 (80)	7/8 (87.5)	8/8 (100)
Genetic and developmental disorders	7/10 (70)	8/10 (80)	7/7 (100)
Neuro‐oncology/neuro‐oncological disorders	7/10 (70)	8/8 (100)	7/7 (100)
Headaches and pain disorders	6/10 (60)	7/8 (87.5)	4/5 (80)
Movement disorders	6/10 (60)	9/10 (90)	6/6 (100)
Neurology of systemic disease	6/10 (60)	7/8 (87.5)	6/6 (100)
Vascular disorders	6/10 (60)	8/10 (80)	6/6 (100)
Behavioural and neurocognitive disorders	5/10 (50)	6/10 (100)	6/6 (100)
Cranial nerve palsies	1/2 (50)	1/1 (100)	1/1 (100)
Neuroinfectious disease	5/10 (50)	4/8 (50)	5/5 (100)
Neuromuscular diseases/disorders	5/10 (50)	6/10 (60)	5/5 (100)
Neuroimmunological and paraneoplastic disorders of the CNS and demyelinating disorders	3/10 (30)	6/7 (85.7)	3/3 (100)
Total	**128/188 (68.1)**	**139/164 (85)**	**123/128 (96.1)**

Abbreviations: AI, artificial intelligence; CNS, central nervous system.

**TABLE 2:** AI chatbot versus peers’ success rate.

Category	AI chatbot success rate (%)	Peers’ success rate, mean (%, SD)
Normal structure process and development through life cycle	100	60 (34.6)
Anatomy/localization	100	70 (28.3)
Ethics/professionalism	100	66.7 (41.2)
Brain and spinal trauma	90	76 (17.1)
Sleep disorders	90	82 (19.3)
Brainstem and spinal cord syndromes	80	78 (21.5)
Epilepsy	80	66 (22.2)
Metabolic diseases, nutritional deficiency states and disorders due to toxins, drugs and physical agents	80	80 (25.9)
Neuro‐ophthalmology/neuro‐otological disorders	80	67 (25.4)
Psychiatric disorders	80	76 (29.5)
Autonomic nervous system disorders	77.8	72.2 (27.3)
Genetic and developmental disorders	70	64 (30.6)
Neuro‐oncology/neuro‐oncological disorders	70	63 (31.6)
Headaches and pain disorders	60	77 (28.3)
Movement disorders	60	76 (19)
Neurology of systemic disease	60	73 (18.3)
Vascular disorders	60	73 (21.1)
Behavioural and neurocognitive disorders	50	76 (27)
Cranial nerve palsies	50	60 (42.4)
Neuroinfectious disease	50	60 (35.6)
Neuromuscular diseases/disorders	50	56 (33.7)
Neuroimmunological and paraneoplastic disorders of the CNS and demyelinating disorders	30	53.5 (34.8)
Total, mean (%, SD)	**71.3 (19.1)**	**69.2 (12.0)**

Abbreviations: AI, artificial intelligence; CNS, central nervous system.

In secondary end‐point analysis, when assessing success rate per category, the performance of the AI chatbot showed discrepant results across different domains. The categories with the highest scores were (1) normal structure process and development through life cycle (100%), (2) anatomy/localization (100%) and (3) ethics/professionalism (100%) versus 60%, 70% and 67% in peers, respectively. Other categories with high success rates were (5) brain and spinal trauma (90%), (6) sleep disorders (90%) and (4) autonomic nervous system disorders (77.8%), (8) epilepsy, (9) metabolic diseases, nutritional deficiency states and disorders due to toxins, drugs and physical agents, and (10) neuro‐ophthalmology/neuro‐otological disorders (80% each). There were categories with lower performance, however, like (19) behavioural and neurocognitive disorders, (20) cranial nerve palsies, (21) neuroinfectious disease and (12) neuromuscular diseases/disorders (50% each) and, in particular, (22) neuroimmunological and paraneoplastic disorders of the central nervous system and demyelinating disorders, with a success rate of 30%. For these categories, human peers' success rates were 76%, 60%, 60%, 56% and 53%, respectively.

Amongst 164 questions without an explicit diagnosis in the clinical vignette, the AI chatbot could identify the correct diagnosis in 139 (85.0%). Failing the correct diagnosis was the main reason for missing the correct answer in the primary end‐point, corresponding to 42% of the cases (25/59 wrong answers). Other domains of incorrectness were treatment/medication management (25.6%); clinical signs (most common neurological finding, topographical diagnosis based on examination) (8.6%); complementary diagnostic tests (most common findings, possible test contaminators) (6.8%); prognosis (5.2%) or other (such as risk factors and epidemiology) (10.3%).

In correct justification analysis, the AI chatbot provided an adequate explanation for its correct answers in 123 out of 128 cases (96.1%).

## DISCUSSION

### AI chatbot performance overview

The difference in performance between the AI chatbot and human experts was not statistically different, meaning that ChatGPT, on average, performed on a par with human neurologists/neurology residents (estimated in 1500 users) in what concerns correctly answering complex multiple choice questions through the interpretation of neurology‐related clinical vignettes. This is notable because it suggests that, at least in the context of these questions, LLM‐based AI models can match human performance.

Despite these results, AI chatbot has shown variable success across different categories, ranging from 30% to 100% (see Table 1). It performed better in categories such as normal structure process and development through life cycle, anatomy/localization and ethics/professionalism. It should be noted that these were categories with particularly few questions (three, one and three respectively), as was the case for cranial nerve palsies with only two questions available, making them prone to bias. Therefore, care should be taken in drawing conclusions about AI performance in such domains. It was interesting to note, however, the high success rate for ethics/professionalism, as ethical problems often require whole‐picture analysis of difficult and nonlinear scenarios—although only three questions were available. In categories where AI chatbot showed a success rate above 60%, it was consistently better than human experts. On the other hand, it performed worse than humans in categories with a success rate ≤60%, particularly in neuroimmunological and paraneoplastic disorders of the central nervous system and demyelinating disorders (30% vs. 53.5%). The variability in performance across different categories may be attributed to differences in complexity of the various neurological fields, to particularly challenging conditions addressed by the selected questions and/or limitations and scarcity of the training data. Categories with lower performance of the AI chatbot like neuromuscular diseases/disorders and neuroimmunological and paraneoplastic disorders of the central nervous system and demyelinating disorders can be particularly challenging due to the fast evolution and growing knowledge in pathophysiology and available treatments as well as recent updates in classification systems. Not only are data in the AI chatbot outdated in some fields (the current publicly available version has information until September 2021), but also access to reliable scientific literature is frequently restricted to paid platforms such as scientific journals, making it impossible for AI chatbot to access the knowledge necessary to correctly answer some questions. Further refinement and optimization of the AI chatbot's algorithms and training processes are essential to enhance its performance across all neurology domains.

### Diagnostic capability and answer justification

The results demonstrate that the AI chatbot is able to infer the correct diagnosis from clinical information in a significant number of scenarios (85%), which is a crucial step in clinical practice to make assertive decisions related to treatments, selecting appropriate examinations and defining prognosis. Although knowing the diagnosis was not sufficient to correctly answer the multiple choice question, it seemed a necessary condition, as shown by the fact that it failed all questions where it did not reach a correct diagnosis. Actually, this was the main reason for failure, in 42% of cases.

By asking ChatGPT to ‘justify’ its correct answers, an extra layer of validity was also added to this investigation. LLMs like ChatGPT reach an answer by associating words in a neural network architecture that was pre‐trained to then choose the most probable text continuation as an output [14]. They do not actually ‘know’ the concepts related to the diseases. By asking for the justification, it is believed that it is possible to analyse the process of ‘reasoning’, trying to clarify and confirm if it uses correct information to then generate its answer, thus making the results more trustworthy and valuable—a confirmatory test—and to demonstrate that it is capable of sequential reasoning through these complex medical questions. It provided an adequate justification in the vast majority of answers, suggesting that it offers explanations that are appropriate and potentially valuable in clinical decision‐making.

### Study limitations

It is important to acknowledge that the study has certain limitations. First, the performance of the AI chatbot was evaluated using a specific dataset and may not be representative of real‐world clinical scenarios. Secondly, there is also variability in what concerns the difficulty inherent to the questions across the different categories, as they were not made by the same person and were randomly chosen across a set of questions. This may have been in part responsible for the variability in performance across different categories, as discussed above. Thirdly, it should be highlighted that answers were only based on interpretation of human language and never on images, so no extrapolations can be made about AI chatbot performance in scenarios where images are crucial to interpretation or reasoning. Fourthly, different prompts including more information, such as defining the role as neurologist, could have been used. Additionally, methodological limitations come from the fact that the answers from the AI chatbot were not individually compared to a peer. Instead, using a mean percentage of correct answers by multiple peers makes statistical tests more fragile in inferring definite conclusions about differences in performance between AI chatbots and human experts.

Lastly, the study focused solely on neurology questions, so AI chatbot's performance in other medical specialties remains to be explored.

### The role of AI in clinical practice and future implications

It must be highlighted that analysing clinical vignettes is somehow artificial compared to a physician's clinical practice. The medical role implies collecting clinically relevant information, filtering out some details and asking the right questions in an imperfect, sometimes desperate, plight scenario with limited time. Moreover, clinicians attend patients who are not always cooperative or able to describe their symptoms, when the main symptom is, quite often, just one more piece in a large puzzle that is slowly uncovered throughout the various consultations and follow‐ups. Beyond diagnosing diseases and treating them according to the latest guidelines, the physician's mission also includes managing the patient as a whole, which often encompasses social and emotional support, dealing and managing expectations and creating empathy/healthy patient−physician relationships based on trust. In this process, decisions about disease management are often nonlinear and may not follow strict guidelines or the apparent best clinical practice.

Concerning the above, whilst the AI chatbot shows promise as a valuable tool in the field of neurology, it is important to emphasize that it should not replace human expertise. Otherwise, the integration of AI technologies should remain being investigated as a potential complementary approach, supporting clinicians in their decision‐making process in order to improve care.

GPT‐3.5 was used for this paper, although by the study's end a new version had already been released (GPT‐4) and, it is hoped, many others will do so in the not too distant future. It is hence a limitation for the present study to use GPT‐3.5 and not GPT‐4 which, according to OpenAI, is trained with around 100 trillion parameters (vs. 175 billion in GPT‐3.5), giving more accurate and coherent responses. It is also capable of accessing the internet meaning that its information is up to date and multimodal, capable of analysing text, images and voice. By using GPT‐4, it was expected to perform even better. It paves the way for future studies comparing these different softwares.

Although overwhelming, it must be taken note that the pace at which technology—namely AI—evolves and advances is still, apparently, exponential. With the methodology used in this paper there is confidence that our work can be replicated and refined in future studies to test the utility and reliability of LLMs in clinical practice.

At present, AI−physician interaction is already possible but requires a specialized neurologist with refined critical thinking and accurate reasoning to validate the output. As they are scalable and tendentially cheaper, enhanced LLMs and other AI technologies have the potential to have a global reach, including developing countries with scarce access to differentialized medical care. For this reason, it is therefore important that the value is recognized and credit is given to companies such as OpenAI that provide these tools for public use, free of charge it is hoped, despite the high financial efforts to develop and maintain them.

## CONCLUSIONS

The results of this study suggest that the LLMs (in our case GPT‐3.5) show promising capabilities in analysing extensive clinical vignettes, interpreting them and providing a diagnostic hypothesis. Less commonly, but also with remarkable accuracy, they are able to answer two‐step questions inquiring about the most effective tests, the best treatment, prognosis and additional considerations reflected in the multiple choice questions. The success rate of AI answers was similar to neurologists/neurology residents with a mean success rate of 71.3% (chatbot) versus 69.2% (peers). This study suggests the potential of integrating AI technologies as a complementary approach to human expertise to support clinicians in their decision‐making process and enhance patient care. Despite being far from being completely reliable, they represent a promising tool. Examining the performance of these models in challenging medical questions can support and encourage further benchmarking in the medical community, in order to learn more about their utility in clinical practice.

## AUTHOR CONTRIBUTIONS

**Ângelo Fonseca:** Conceptualization; investigation; writing – original draft; methodology; formal analysis; project administration; resources; data curation. **Axel Ferreira:** Investigation; methodology; validation; writing – review and editing. **Luís Ribeiro:** Writing – review and editing; validation. **Sandra Moreira:** Writing – review and editing; validation. **Cristina Duque:** Writing – review and editing; validation.

## CONFLICT OF INTEREST STATEMENT

The author and coauthors report no conflict of interest.

## ETHICAL APPROVAL

Since data consist in generical clinical vignettes and in percentages of correct answers provided by AAN and there is no access to the identities of the participants (the percentages are provided to all ‘Question of the Day’ app users) who are therefore anonymous (as they are part of the AAN database, not ours), no ethics approval was sought. Furthermore, the AI chatbot is freely accessible to the public.

## Data Availability

The data that support the findings of this study are available from the corresponding author upon reasonable request.
